# New Appliances and Things Medical

**Published:** 1904-07-30

**Authors:** 


### NEW APPLIANCES AND THINGS MEDICAL.

[We shall be glad to receive, at our Office, 28 & 29 Southampton Street, Strand, London, W.C., from the manufacturers, specimens of all new preparations and appliances which may be brought out from time to time.]

### PROTENE.

(The Protene Co, 36 Welbeck Street, London.)

This substance, as is well known, consists practically of pure casein, and was one of the earliest preparations of this substance made on the commercial scale and put on the market. In addition to the pure product itself, the company now makes several food preparations, the proteid worth of which is greatly enhanced by the addition, in varying quantities, of protene. There can be no doubt as to the value of this preparation, nor as to the value of the foods made with it, when a diet is required rich in unirritating proteid.

### DUNVILLE'S SPECIAL LIQUEUR WHISKY.

(Dunville and Company, Limited, Belfast.)

We have received a sample of this whisky. The whisky seems a thoroughly good one, and gives chemical evidence of at least largely consisting of genuine pot-still whisky. Its flavour is pleasant, and indicates full maturation. We think it is to be recommended.

